# Dose-Dependent Alterations in Lung Immune Subpopulations in Influenza a Virus Infection

**DOI:** 10.3390/ijms27177522

**Published:** 2026-08-22

**Authors:** Tatiana Betáková, Miriam Mladá, Karin Donátová, Jana Jakubíková

**Affiliations:** 1 Department of Microbiology and Virology, Faculty of Natural Sciences, Comenius University in Bratislava, 842 15 Bratislava, Slovakia; donatova3@uniba.sk; 2 Biomedical Research Center, Institute of Virology, Slovak Academy of Sciences, 845 05 Bratislava, Slovakia; miriam.mlada@savba.sk; 3 Department of Tumor Immunology, Cancer Research Institute, Biomedical Research Center, Slovak Academy of Sciences, 84505 Bratislava, Slovakia; jana.jakubikova@savba.sk

**Keywords:** influenza virus, cytokine, receptor, immune cells

## Abstract

This study aimed to characterize the modulation in immune cell subpopulations in murine lungs following influenza A virus (IAV) infection, assessing the effects of infectious dose, viral adaptation, and NS1 expression. Immune cell subsets were profiled by surface receptor expression using multiparametric flow cytometry with a 10-antibody immunophenotyping panel. Neutrophils expressing Ly-6G were significantly increased in the lungs following lethal-dose infection with IAV, independently of NS1 expression; in contrast, lethal-dose infection with all viruses reduced CD163^+^ and F4/80^+^ neutrophil subpopulations. Lethal-dose infection increased pulmonary CD68^+^ macrophages while decreasing CD163^+^, CD193^+^, and F4/80^+^ macrophage subsets, as well as F4/80^+^ myeloid cells, by day 3 post-infection; these reductions were independent of NS1 expression and infectious dose. Following lethal-dose IAV infection, NK cells exhibited upregulation of IL-23R^+^ and IL-12Rβ2^+^ subsets, while the CD193^+^ NK subpopulation was decreased on day 3 post-infection. Profiling of NKT cells revealed an expansion of the IL-12Rβ2^+^ NKT subset on day 3 post-infection. Adaptive immune profiling of lung CD4^+^ T cells revealed a selective increase in Th1-like cells (IL-12Rβ2^+^ CD4^+^) after WSN infection, a marked reduction in Th2-like cells (CD193^+^ CD4^+^) following infection with IAV regardless of NS1 status or dose, and an expansion of CD4^+^NK1.1^+^ cells only after lethal-dose infection. Immune cell subset frequencies were comparable between infections with NS1-expressing and wild-type viruses; NS1 expression did not alter subset composition, whereas the infection dose modulated their abundance. These findings expand our understanding of the subpopulation of immune cells and their possible role in influenza virus pathogenesis.

## 1. Introduction

Innate and adaptive immunity orchestrate temporally and functionally distinct cellular programs to control pathogens. Innate effector cells, including neutrophils, macrophages, natural killer (NK) cells, and NKT cells, provide rapid, antigen-independent detection and clearance of viruses and infected cells during the early phase of infection, prior to the activation and clonal expansion of antigen-specific T and B lymphocytes [1]. Populations of immune cells can be distinguished by the expression of specific surface markers [2]. The presented antigen, cytokines, transcription factors, antigen-presenting cells (APCs), and signals from the environment determine the differentiation of specific cell subpopulations [3]. It has long been assumed that immune cells form a homogeneous population of cells that perform various antibacterial and antiviral functions. Various populations of circulating immune cells have been identified based on cell-surface markers, localization, maturity, functions, and other discrete parameters [4,5,6,7,8,9,10]. Heterogeneity of immune cells is associated with their distinct effector activities. Immune cells communicate with each other through direct cell–cell interaction and soluble factors, mainly cytokines [11]. A pathophysiological network created by the subpopulations of immune cells determines the development of diseases and spread of pathogens.

The influenza virus imposes a substantial global health burden, causing an estimated 1 billion infections annually, of which 3–5 million progress to severe disease and approximately 290,000–650,000 result in death every year [12]. Influenza A virus (IAV) belongs to the family *Orthomyxoviridae*. The genome contains eight segments of single-stranded RNA and encodes up to 18 proteins. A multifunctional non-structural NS1 protein contributes to influenza virulence by regulating viral replication and inhibiting host antiviral responses during infection. The NS1 protein is composed of three functional domains. The N-terminal RNA-binding domain binds double-stranded RNA (dsRNA), inhibits the interferon (IFN) response, and plays an essential role in the translation of viral mRNA [13]. The short linker region between the N-terminal and effector domains interacts with more than 50 host proteins, can bind CPSF30, and inhibits recognition of polyadenylation signals by the CPSF complex at the 3′ end of mRNAs during transcription, thereby preventing cleavage of immature pre-mRNAs and recruitment of the poly(A) polymerase to synthesize the poly(A) tail [14]. The effector domain inhibits the synthesis of type I interferons and pro-inflammatory proteins by preventing activation of the interferon regulatory transcription factor IRF3, nuclear factor-κB (NF-κB), and cJun/ATF2 [15,16]. The C-terminal domain of the NS1 protein further contributes to inhibition of IFNs and cytokine production in infected cells by regulating apoptosis and activating the PI3K signaling pathway. IAVs with mutated or deleted NS1 protein are associated with increased virulence and pathogenicity [17,18]. However, the key finding is that deletion of NS1 paradoxically increases lung inflammation despite reducing viral replication. This occurs because NS1 normally suppresses type I interferon (IFN) and pro-inflammatory cytokine responses; without it, the host mounts an excessive innate immune response (cytokine storm) that causes immunopathology [15,19,20].

In our study, we examined the effects of IAV on immune cells by characterizing the activation of innate and adaptive immune cell subpopulations in the lungs of infected mice. Immune populations were defined based on surface marker expression using a 10-color antibody panel for multiparameter flow cytometry, and we evaluated how infectious dose, viral adaptation, and the NS1 protein influenced their activation.

## 2. Results

### 2.1. Differences Between Non-Lethal and Lethal Infection in Balb/C Mice

A/WSN/33 (WSN) and NS1-truncated (NS80) viruses were prepared using a reverse genetic system. The NS80 virus without effector and C-domains possesses only the first 80 amino acids of the NS1 protein and is attenuated in vitro. Despite this, the NS80 virus is more pathogenic than the control WSN virus because the inflammatory signaling pathways are not inhibited by the NS1 protein. Adaptation of the NS80 virus through the lung passage resulted in increased replication of the NS80ad virus in both the lungs and Vero cells, and the virus was no longer attenuated [21].

**Figure 1:**
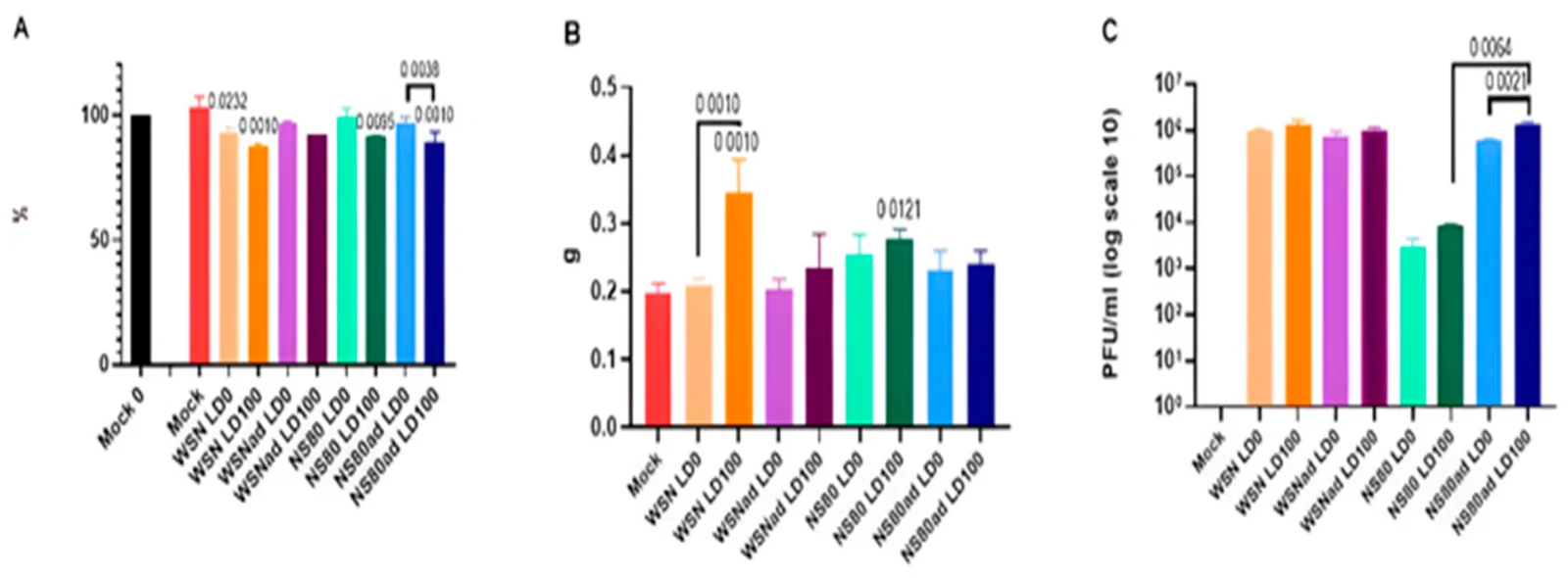
(**A**) The percentage body weight loss relative to the initial weights (day 0) was recorded on the 3rd day p.i. (**B**) The weight of the lungs was recorded on the 3rd day p.i. (**C**) The virus titer was determined in the lung tissue homogenates as described in the Materials and Methods. Mock represents noninfected mice on the third day (Mock). The results represent the mean of two independent experiments (*n* = 4) and are presented as the mean ± SD. Statistical significance was calculated between the control group (Mock) and mice infected with WSN, WSNad, NS80, and NS80ad; between groups infected with LD0 and LD100; between WSN and WSNad, NS80 and NS80ad; between WSN and NS80; and between WSNad and NS80ad. Data were statistically evaluated using one-way ANOVA and post hoc Tukey’s HSD test; *p* < 0.05 is statistically significant; *p* < 0.01 is very statistically significant; *p* < 0.001 is extremely statistically significant.

Balb/c mice were infected with non-lethal and lethal doses of influenza viruses. Some mice infected with LD(100) began to die or had to be euthanized on the fourth day post-infection. Therefore, we conducted all experiments on the third day after infection. The mice were euthanized via cervical dislocation on the third day post-infection, after which lungs were collected for analysis. Mice infected with a non-lethal dose exhibited mild symptoms of the disease, such as ruffled fur and lethargy. In contrast, mice infected with a lethal dose showed more severe signs, including ruffled fur, lethargy, tremors, loss of appetite, and noticeable weight loss (Figure 1A). The weight of the lungs taken from mice infected with a lethal dose of WSN and NS80 viruses was higher than that of mouse lungs after non-lethal infection (Figure 1B). The viral titer was determined in lung homogenates. The virus titer was increased in the lungs of mice infected with a lethal dose of the virus. Significant differences in viral replication were detected, especially after infection with the adapted WSN and NS80 viruses. The virus titer was also notably increased in the lungs of mice infected with NS80ad viruses compared to the non-adapted NS80 viruses (Figure 1C).

### 2.2. Histological Changes in the Lungs of Mice Infected with a Lethal Dose of IAV

**Figure 2:**
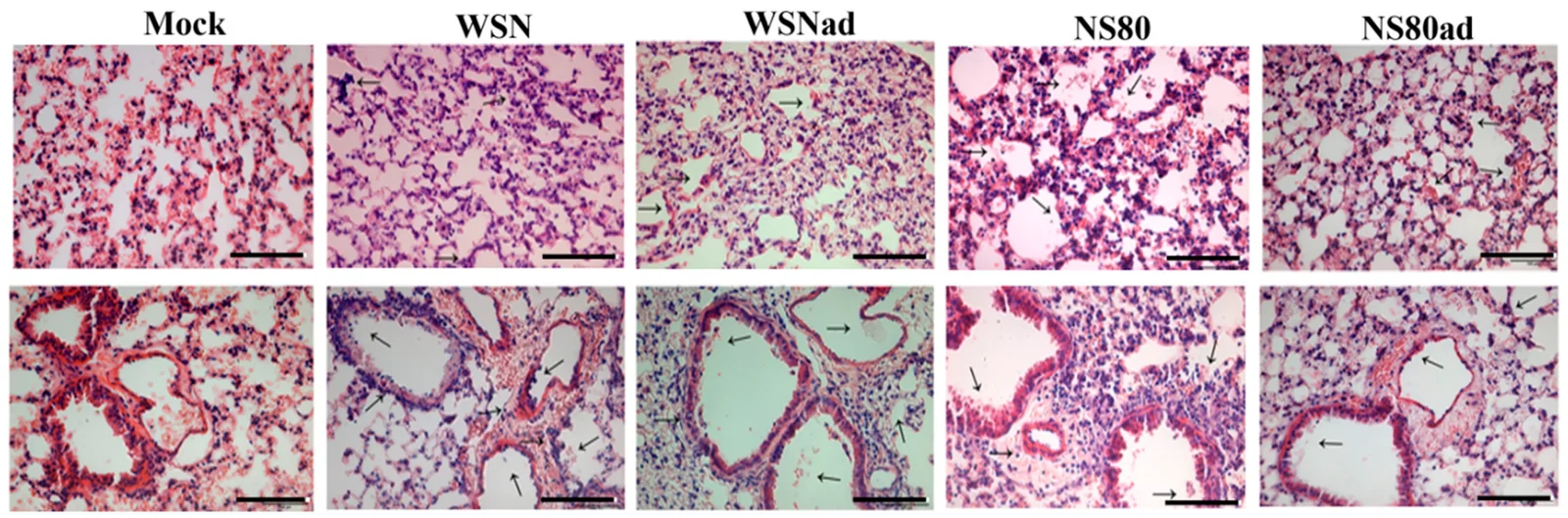
Histopathology of lung tissues. Balb/c mice were infected with lethal doses of WSN, WSNad, NS80, and NS80ad viruses. The lungs were harvested and fixed on the 3rd day p.i. Mock represents samples from noninfected lungs. Sections were obtained using a microtome. Hematoxylin and eosin staining was performed on lung sections from mock (noninfected), WSN-, WSNad-, NS80-, and ND80ad-infected mice. The necrotic lesions are labeled with arrows. Scale bars: 100 µm.

Histological examination of lungs from mice infected with a lethal dose of avian influenza viruses revealed hyperplasia of type II pneumocytes (Figure 2). The epithelium of the terminal bronchioles was visibly thickened, and the inner surface was smooth. In contrast, the ciliated epithelium in small bronchioles tended to shed. Furthermore, the basal membrane of the bronchioles contained a minimal number of basal cells. The infection caused frequent diffuse alveolar damage (DAD) in the lungs, leading to an overall morphological change in the lung structure. Massive eosinophilic inflammation was detected around small bronchioles, and the smooth muscle was thickened. The basement membrane and smooth muscle were significantly thicker after infection with the NS80 virus. The effect of the shortened NS1 protein was also evident in mucus formation in the bronchioles. Alveoli had atypical morphology, exhibiting smooth, predominantly round formations.

### 2.3. Biological Pathways Associated with the NS1 Protein and Viral Adaptation

**Figure 3:**
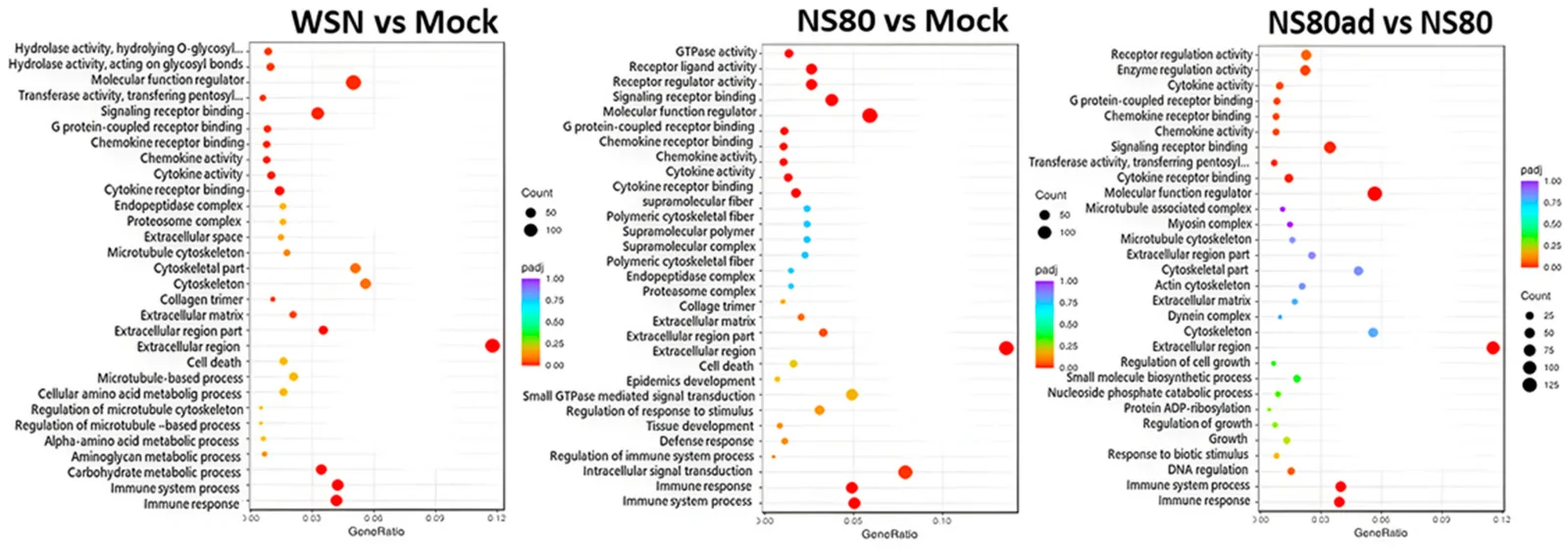
GO enrichment analysis of dysregulated proteins after infection with IAV, comparing noninfected lungs with lungs infected with WSN or NS80 virus and comparing protein dysregulation after infection with non-adapted and adapted NS80 virus. The abscissa in the graph is the ratio of the number of differentially expressed genes to the total number of differentially expressed genes in the GO term; the ordinate is the GO term with a higher rich factor, indicating greater enrichment. The color of the dots represents the enrichment score [−log_10_(*p*-value)], where purple indicates high enrichment, and red indicates low enrichment. Dot size corresponds to the number of proteins in the respective GO terms, with larger dots indicating a greater number of proteins.

To analyze the effects of NS1 loss and adaptation on gene expression after infection, we performed RNA sequencing in the lungs of mice infected with non-adapted and adapted viruses as previously described [21]. To study biological pathways associated with each specific virus, gene ontology (GO) enrichment analysis was carried out, and the most significant results are displayed in Figure 3. GO enrichment analyses showed that the functions of differentially expressed genes in the lungs infected with WSN and NS80 viruses were mainly involved in cytokine activity, regulation of immune response, defense response, and intracellular signal transduction. In contrast, adaptation of the NS80 virus changed the expression of genes associated with regulation of cell growth, small-molecule biosynthesis process, molecular function regulation, enzyme regulation activity, growth, immune response, and immune system process.

**Figure 4:**
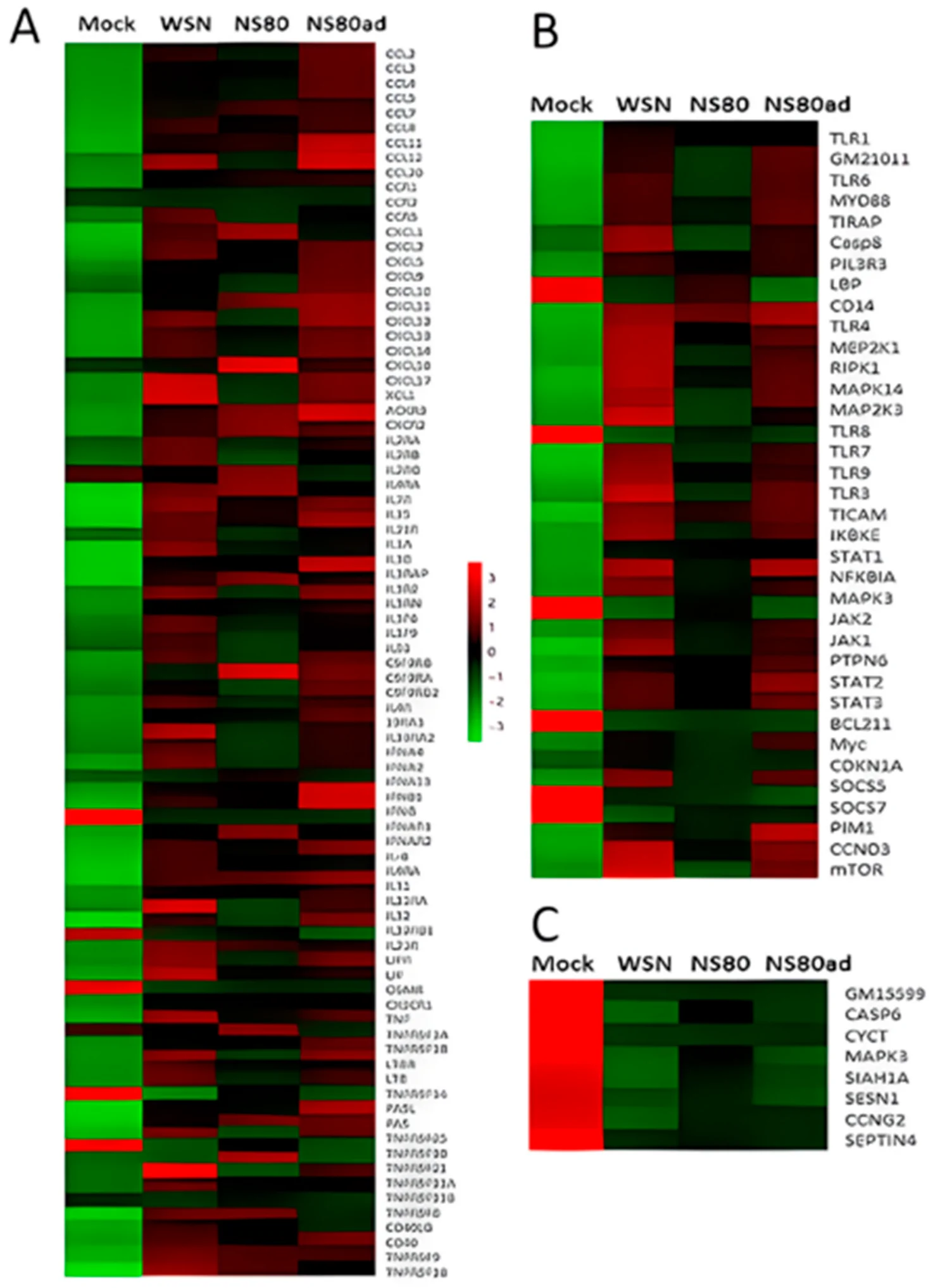
Heatmap of averaged log2(FPKM + 1) values of genes enriched in signaling pathways associated with the cytokine pathway (**A**), Toll-like receptor pathway (**B**), and apoptosis (**C**) in WSN (*n* = 3), NS80 (*n* = 3), and NS80ad (*n* = 3) infected lungs. Red indicates genes with high expression levels, and green indicates genes with low expression levels.

GO analyses were also performed to understand the potential function of differentially expressed genes. The results showed that NS1 protein dysregulated genes were involved in cytokine and Toll-like receptor pathways. Adaptation of the NS80 virus mostly upregulated the genes responsible for the expression of CC, CXC, and CX3CL subfamilies of cytokines, interferons, and TNF family genes (Figure 4A). Dysregulated genes from Toll-like receptor (TLR) pathways were involved in the regulation of the PI3K-Akt signaling pathway, NF-κB signaling pathway, ubiquitin-mediated proteases, MAPK signaling pathway, and JAK-STAT signaling pathway (Figure 4B). The genes involved in the regulation of apoptosis were downregulated after infection with all influenza viruses and were not dependent on the NS1 protein (Figure 4C).

### 2.4. Quantitative Analysis of the Subpopulations of Immune Cells in the Lungs of IAV-Infected Mice

**Figure 5:**
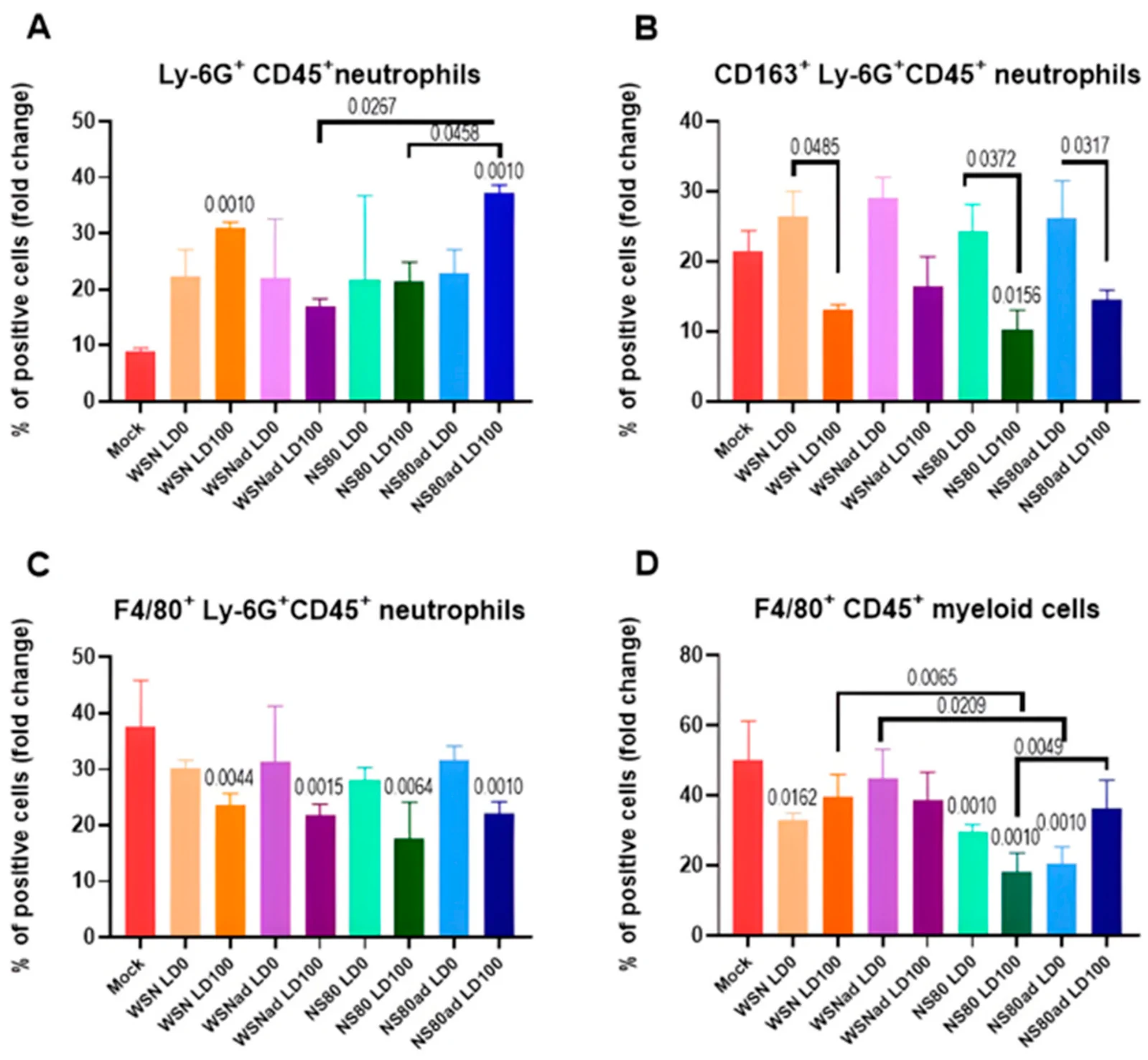
Identification of the neutrophil subpopulations: (**A**) Ly-6G^+^ CD45^+^ neutrophils; (**B**) CD163^+^ Ly-6G^+^ CD45^+^ neutrophils; and (**C**) F4/80^+^ Ly-6G^+^ CD45^+^ neutrophils; (**D**) F4/80^+^ CD45^+^ myeloid cells in the lungs on the third day post-infection. The Balb/c mice were infected with a non-lethal (LD0) or lethal dose (LD100) of IAVs. The immune cells were isolated, labeled with antibodies, and analyzed using a FACS Aria Special Sorter (Becton Dickinson, Mountain View, CA, USA) equipped with multiple laser systems, as described in Materials and Methods. Mocks represent noninfected mice. WSNad and NS80ad were adapted in mice by serial passaging through the lungs. The expression values represent the mean of two independent experiments (*n* = 4) and are presented as the mean ± SD. Statistical significance was calculated between the control group (Mock) and mice infected with WSN, WSNad, NS80, and NS80ad; between groups infected with LD0 and LD100; between WSN and WSNad, NS80 and NS80ad; between WSN and NS80; and between WSNad and NS80ad. Data were statistically evaluated using one-way ANOVA and post hoc Tukey’s HSD test; *p* < 0.05 is statistically significant; *p* < 0.01 is very statistically significant; *p* < 0.001 is extremely statistically significant.

To characterize immune cell responses in murine lungs after IAV infection, multiparametric flow cytometry using a 10-marker panel was performed. Neutrophils play an important role in virus clearance and are the first cells at the site of infection [22,23]. Evaluation of distinct neutrophil subpopulations in the lungs of IAV-infected mice revealed that Ly-6G^+^ neutrophils, defined by Ly-6G expression as a standard murine neutrophil marker, were significantly increased in the lungs following lethal-dose infection with the WSN and NS80ad viruses (Figure 5A). This expansion was independent of NS1 protein expression. In addition, lethal-dose infection with all viruses resulted in decreased proportions of CD163^+^ neutrophil (Figure 5B) and F4/80^+^ neutrophil (Figure 5C) subpopulations. The frequency of F4/80^+^ CD45^+^ myeloid cells was significantly reduced in the lungs on the third day post-infection after non-lethal WSN infection and after both non-lethal and lethal doses of NS80 and NS80ad viruses (Figure 5D).

**Figure 6:**
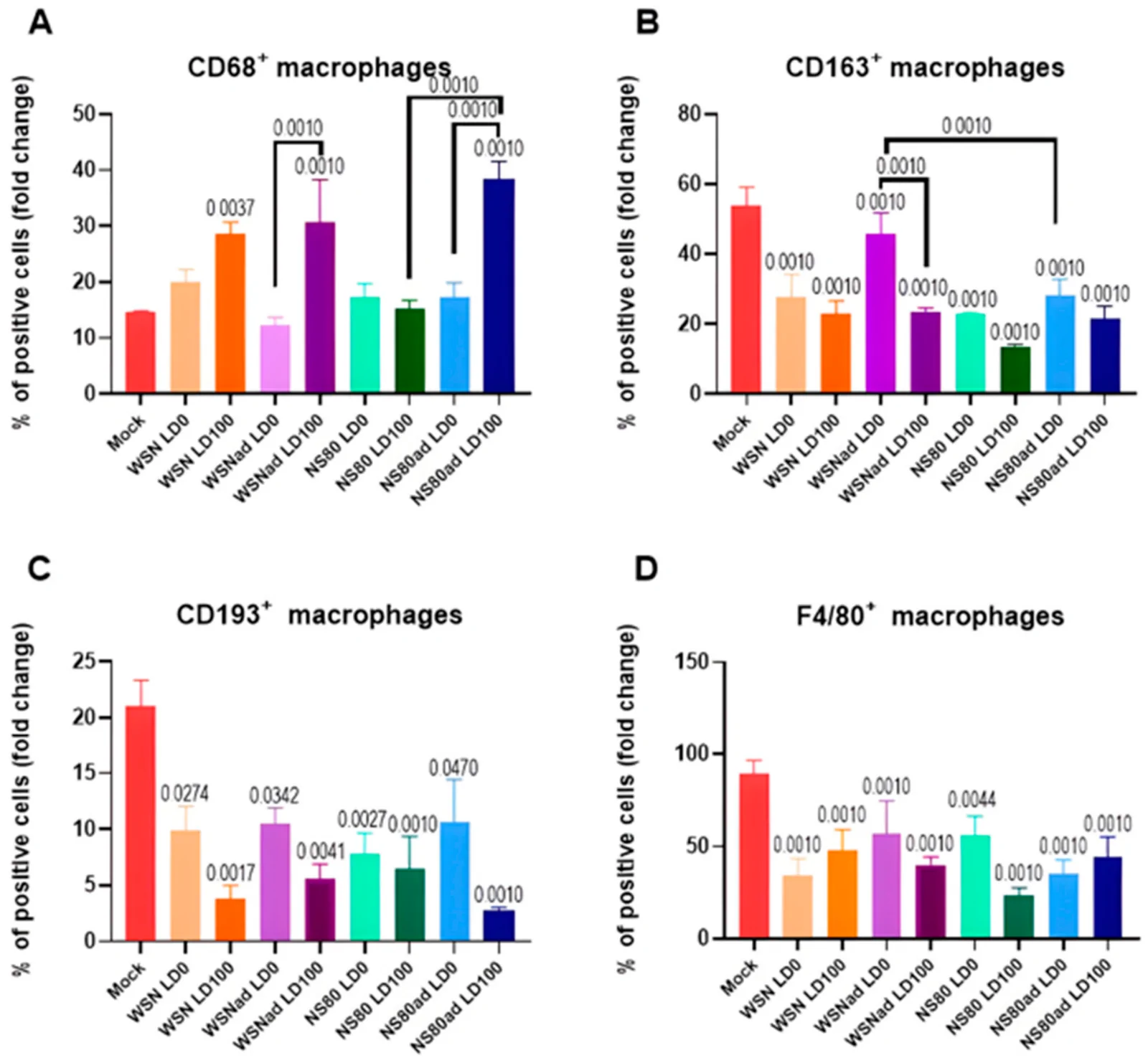
Identification of the subpopulation of (**A**) CD68^+^, (**B**) CD163^+^, (**C**) CD193^+^, and (**D**) F4/80^+^ macrophages in the lungs on the third day post-infection. The Balb/c mice were infected with a non-lethal (LD0) or lethal dose (LD100) of IAVs. The immune cells were isolated, labeled with antibodies, and analyzed using a FACS Aria Special Sorter (Becton Dickinson, Mountain View, CA, USA) equipped with multiple laser systems, as described in Materials and Methods. Mocks represent noninfected mice. WSNad and NS80ad were adapted in mice by serial passaging through the lungs. The expression values represent the mean of two independent experiments (*n* = 4) and are presented as the mean ± SD. Statistical significance was calculated between the control group (Mock) and mice infected with WSN, WSNad, NS80, and NS80ad; between groups infected with LD0 and LD100; between WSN and WSNad, NS80 and NS80ad; between WSN and NS80; and between WSNad and NS80ad. Data were statistically evaluated using one-way ANOVA and post hoc Tukey’s HSD test; *p* < 0.05 is statistically significant; *p* < 0.01 is very statistically significant; *p* < 0.001 is extremely statistically significant.

Macrophages destroy viruses and virus-infected cells through phagocytosis, modulate inflammatory responses, and can also serve as antigen-presenting cells [24]. Infection with a lethal dose of WSN, WSNad, and NS80ad virus resulted in increased levels of CD68^+^ macrophages in the lungs (Figure 6A). Conversely, the proportion of CD163^+^ macrophages decreased in the lungs on the third day after infection with all influenza viruses (Figure 6B). We also examined macrophage subsets expressing CD193 or F4/80; both populations were significantly reduced following influenza virus infection (Figure 6C,D). These reductions were independent of NS1 protein expression and of the infecting virus dose. Overall, the data indicate that lethal-dose influenza virus infection has a greater impact on the reduction in CD163^+^ and CD193^+^ macrophage subpopulations.

**Figure 7:**
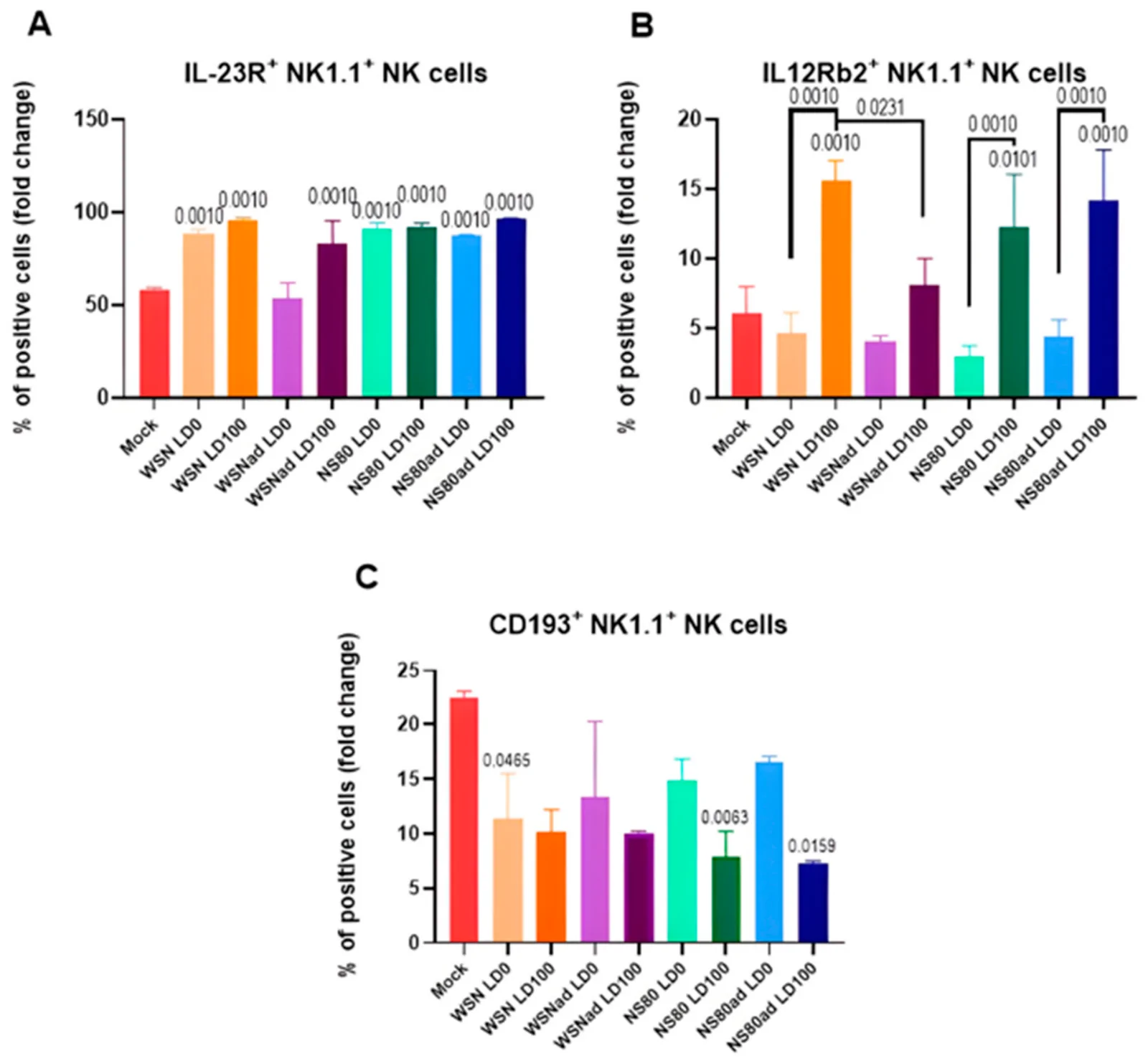
Identification of the subpopulation of (**A**) CD193^+^ NK1.1^+^ NK cells, (**B**) IL/12Rb2^+^ NK1.1^+^ NK cells, and (**C**) IL/23R^+^ MK1.1^+^ NK cells in the lungs on the third day post-infection. The Balb/c mice were infected with a non-lethal (LD0) or lethal dose (LD100) of IAVs. The immune cells were isolated, labeled with antibodies, and analyzed using a FACS Aria Special Sorter (Becton Dickinson, Mountain View, CA, USA) equipped with multiple laser systems, as described in Materials and Methods. Mocks represent noninfected mice. WSNad and NS80ad were adapted in mice by serial passaging through the lungs. The expression values represent the mean of two independent experiments (*n* = 4) and are presented as the mean ± SD. Statistical significance was calculated between the control group (Mock) and mice infected with WSN, WSNad, NS80, and NS80ad; between groups infected with LD0 and LD100; between WSN and WSNad, NS80 and NS80ad; between WSN and NS80; and between WSNad and NS80ad. Data were statistically evaluated using one-way ANOVA and post hoc Tukey’s HSD test; *p* < 0.05 is statistically significant; *p* < 0.01 is very statistically significant; *p* < 0.001 is extremely statistically significant.

Analysis of NK populations revealed changes in three NK cell subpopulations. IL-23R^+^ NK cells were elevated irrespective of dose and NS1 status (Figure 7A), and the subpopulation of IL-12Rb2^+^ NK cells was significantly increased after infection with a lethal dose of all influenza viruses (Figure 7B). On the other hand, all viruses reduced the frequency of the CD193^+^ NK subpopulation in infected lungs (Figure 7C).

**Figure 8:**
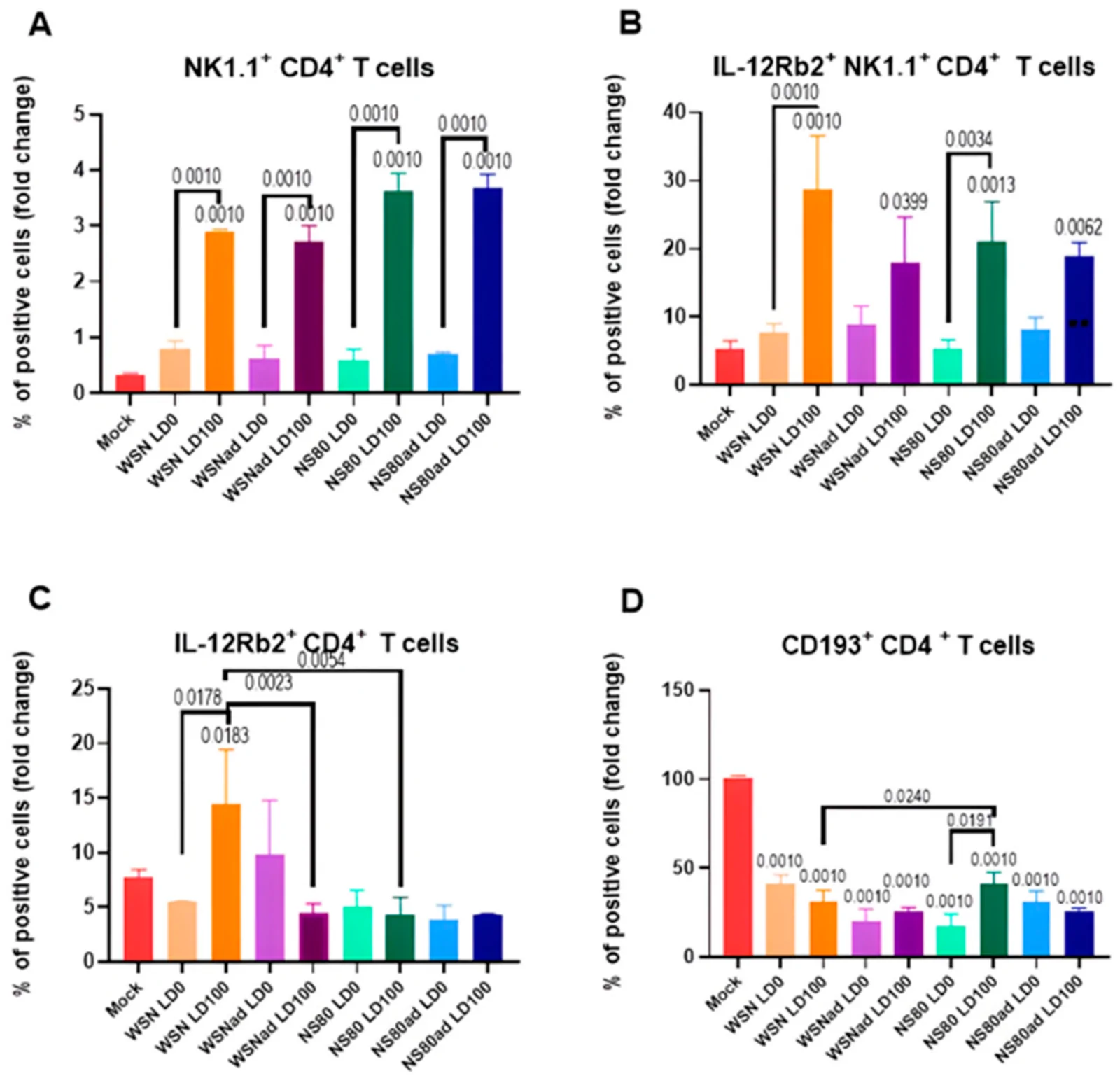
Identification of the subpopulations of (**A**) NK1.1^+^ CD4^+^ T cells; (**B**) IL/12Rb2^+^ NK1.1^+^ CD4^+^ T cells; (**C**) IL/12Rb2^+^ CD4^+^ T cells; and (**D**) CD193^+^ CD4^+^ T cells in the lungs on the third day post-infection. The Balb/c mice were infected with a non-lethal (LD0) or lethal dose (LD100) of IAV. The immune cells were isolated, labeled with antibodies, and analyzed using a FACS Aria Special Sorter (Becton Dickinson, Mountain View, CA, USA) equipped with multiple laser systems, as described in Materials and Methods. Mocks represent noninfected mice. WSNad and NS80ad were adapted in mice by serial passaging through the lungs. The expression values represent the mean of two independent experiments (*n* = 4) and are presented as the mean ± SD. Statistical significance was calculated between the control group (Mock) and mice infected with WSN, WSNad, NS80, and NS80ad; between groups infected with LD0 and LD100; between WSN and WSNad, NS80 and NS80ad; between WSN and NS80; and between WSNad and NS80ad. Data were statistically evaluated using one-way ANOVA and post hoc Tukey’s HSD test; *p* < 0.05 is statistically significant; *p* < 0.01 is very statistically significant; *p* < 0.001 is extremely statistically significant.

The subpopulation of CD4^+^ T cells with the NK1.1 receptor on the surface was significantly increased in the lungs after infection with a lethal dose of the virus (Figure 8A). In addition, profiling NKT cells, which share properties of both T cells and natural killer cells, revealed that the IL-12Rb2^+^ NKT subset was greatly increased in the lungs infected with a lethal dose of influenza virus (Figure 8B).

Adaptive immune profiling of CD4^+^ T-cell subsets revealed that the Th1-like subset (IL-12Rb2^+^ CD4^+^ T cells) was increased only after infection with the lethal dose of WSN virus (Figure 8C). In contrast, Th2-like cells (CD193^+^ CD4^+^ T cells) were significantly reduced in the lungs of mice infected with IAVs regardless of the effect of NS1 or the infectious dose of the virus (Figure 8D).

Overall, lethal-dose IAV infection preferentially expanded pro-inflammatory Ly-6G^+^ neutrophils, CD68^+^ macrophages, IL-12Rβ2^+^ and IL-23R^+^ NK cells, IL-12Rβ2^+^ NKT cells, and IL-12Rβ2^+^ CD4^+^ and CD4^+^ NK1.1^+^ T cells. Concurrently, it depleted select populations including CD163^+^ and F4/80^+^ neutrophils, CD163^+^ and CD193^+^ macrophages, F4/80^+^ CD45^+^ myeloid cells, and CD193^+^ NK and CD4^+^ T cells.

## 3. Discussion

Virus pathogenicity is affected by numerous factors, and the ability to clear the virus and the mortality of the host depend on the initial viral dose [25,26]. Mice infected with a non-lethal dose of the virus showed only mild or moderately severe symptoms of the disease. These mice had slightly ruffled fur, weak tremors, and lost their appetite. From the fourth day onwards, the health condition of these mice improved rapidly, and by the seventh day, there were no evident symptoms of the disease [25]. The mice infected with a lethal dose of the virus were lethargic, had bristled fur, and significantly lost weight. The lungs of mice infected with a lethal dose exhibited larger macroscopic lesions and more pronounced histological changes than those infected with a non-lethal dose [25,27,28]. The viral titer was similar after infection with non-lethal and lethal doses of the non-adapted viruses.

The virulence and pathogenicity of the influenza viruses can be increased by serial lung-to-lung passages. Adaptation increased the replication ability of the NS80 virus by enhancing the ability of NS80ad to inhibit cellular machinery, block apoptosis, and use the cell for its own replication. The adapted WSN and NS80 viruses exhibited significantly increased replication following infection with a lethal dose [21]. Even though the NS80 virus is attenuated, it is much more pathogenic than the wild-type WSN virus [18]. Truncation of NS1 led to reduced replication in vitro and in vivo, but, on the other hand, infection with this virus activated immune signaling pathways and cytokine production to a greater extent than infection with the parental virus [18,29,30]. The NS1 protein also regulates the TLR signaling pathway through various pathways and plays an important role in innate and adaptive immune responses [31,32,33]. We have already shown that WSN and NS80 viruses deregulate the expression of more than 600 genes involved in the regulation of cellular machinery and apoptosis. The NS80 virus specifically deregulates 116 genes, which are mostly associated with the regulation of the immune response [21,30]. GO enrichment analyses showed that adaptation of the NS80 virus resulted in overexpression of cytokines in the lungs of infected mice. While infection with a lethal dose of the influenza virus uncontrollably increases the expression of interleukins and CC chemokines, the NS1 protein controls the expression of pro-inflammatory cytokines, interferons, and some CC- and CXC-chemokines [18,26]. The profile of cytokines was completely altered after infection with the adapted virus compared to infection with the non-adapted virus. This leads us to the conclusion that elevated pro-inflammatory cytokines and chemokines with various activities led to the development of hypercytokinemia, also known as a “cytokine storm”, and this is associated with poor clinical outcome and pathogenesis during influenza infection [34,35].

In the lungs infected with IAVs, we detected subpopulations of immune cells, including neutrophils, NK cells, NKT cells, macrophages, eosinophils, and Th lymphocytes. Neutrophils, like the first immune cells, migrate to the site of infection, where they play an important role in recognizing the virus. Currently, approximately 370 CD markers on the surface of neutrophils are defined. However, based on current knowledge, it is not yet possible to identify “good” or “bad” neutrophils [36]. We examined three subpopulations of neutrophils in the lungs of infected mice based on surface markers Ly-6G^+^, CD163^+^, and F4/80^+^, the former of which is a typical marker on the neutrophil surface [37,38]. We found that the level of the Ly-6G^+^ neutrophil subpopulation was significantly increased, particularly after infection with WSN (LD0) and NS80ad (LD100). It is known that excessive activation and migration of neutrophils to the site of inflammation may contribute to the worsened pathogenesis of influenza disease [23,39,40,41]. In the lungs, we detected subpopulations of neutrophils that expressed not only the Ly-6G marker but also surface markers typical for macrophages (CD163^+^) and myeloid cells (F4/80^+^). CD163^+^ neutrophil populations have not yet been described in connection with mice and with IAV infection. It was found that the expression of CD163 on neutrophils was elevated in critically ill neonates and children with systemic inflammatory response syndrome with sepsis [42]. In our study, the levels of these neutrophils were reduced after infection with a lethal dose. CD163 is a scavenger receptor typically expressed on macrophages, especially on anti/inflammatory M2 macrophages [43]. Based on our results, we can hypothesize that CD163+ neutrophils play a positive role in the elimination of infected cells. F4/80 antigen is usually used as a differentiation marker of tissue macrophages in mice and was not detected on neutrophils [44,45]. Because the CD163^+^ and F4/80^+^ subpopulations of neutrophils were significantly reduced, especially by lethal infection with the influenza virus, we suggest that the lack of these cells could play an important role in the clearance of tissue infected with the virus. It would be interesting to find out how these subpopulations of neutrophils cooperate with other immune cells in the elimination of the virus and infected cells, and how a reduced number of these cells affects the deterioration of the condition in infected mice.

After infection with IAV, macrophages were polarized to classically activated CD68^+^ and alternatively activated CD163^+^ macrophages. The proportion of CD68^+^ macrophages increased in the lungs infected with a lethal dose of IAV, except in the case of the NS80 virus. On the other hand, the number of CD163^+^ macrophages decreased independently of the viral infection dose and NS1 protein. The increased number of M1 macrophages has a protective effect on the lungs during infection [46]. CD163 is highly expressed on resident tissue macrophages in vivo, where it acts as an inducer of local inflammation [47].

Two other distinct macrophage subpopulations expressing CD193^+^ and F4/80^+^ were detected. The frequency of these cells was significantly decreased after infection with all influenza viruses, and this reduction was not dependent on the infection dose or NS1 protein. The vast majority of tissue-resident macrophages express F4/80. A high number of F4/80 marker copies were detected on large peripheral macrophages. Previous studies have shown that after infection or inflammation, large peripheral macrophages disappear and are replaced by small peripheral macrophages, which express a small number of F4/80 markers [48,49]. This finding corresponds with our results, where the number of F4/80^+^ macrophages was significantly reduced after infection with IAV. CD193 is not a constitutive macrophage marker, but can be upregulated under specific inflammatory conditions [50,51]. CD193^+^ macrophages often expressed other receptors on their surface that influenced their function. Therefore, it is difficult to predict the function of the CD193^+^ macrophages we detected. The CD193 receptor plays a role in inflammation, migration of macrophages towards eotaxin, macrophage polarization, fibrosis, and wound healing via eotaxin signaling [51,52,53].

The activity of NK cells is tightly regulated by multiple germ-line-encoded activating and inhibitory receptors. The ligands for these receptors can activate or inhibit NK cell responses and alter their cytotoxic activity. NK cells also produce cytokines that can affect innate and adaptive immunity [54]. Cytotoxic reactions are triggered either classically via activation and inhibitory receptors, or by subpopulations of NK cells that also have, in addition to the typical NK1.1 marker, IL-12Rb2, CD193, or IL-23 receptors. The lethal dose did not affect the overall frequency of NK cells with the NK1.1 marker in the lungs of mice after infection with influenza viruses. However, it was interesting that NK cells expressing the IL-12Rb2 marker were significantly increased in the lungs only after infection with a lethal dose of IAV. In previous studies, we found that the level of IL-12 was significantly increased in the lungs of mice infected with a lethal dose of the highly virulent virus A/PR/8/34 (H1N1) [26]. Pro-inflammatory cytokine IL-12 is known globally to promote NK cell activation and cytotoxicity and has global effects on the immune system. Many hematopoietically derived cells can express the IL-12R [4]. Our results suggest that an increased level of NK cells with the IL-12Rb2 receptor can be closely associated with the increased expression of IL-12. IL-23 is structurally related to IL-12. IL-23 is critical for protection against challenges with several extracellular pathogens [55,56,57]. A paradigm has emerged in which the IL-23/IL-17 axis enhances neutrophil production and recruitment and promotes epithelial cell barrier function and repair that is required for resistance to extracellular pathogens [58]. The level of IL-23R^+^ NK cells increased in the lungs after infection with IAV and did not depend on the dose or NS1 protein. We assume that this subpopulation of NK cells plays an important role in virus clearance. The receptor CD193 can be expressed on immune cells such as T cells, neutrophils, B cells, basophils, etc. [8,9,10]. Previous studies have shown the important role of the CCR3 (CD193) pathway in the activation of eosinophils, Th2 cells, and mast cells in vivo and have also demonstrated increased CD193 expression in allergic airway diseases [59]. We detected the CD193 receptor on NK cells. However, the proportion of these cells decreased in the lungs, especially after infection with the WSN virus and a lethal dose of NS80 and NS80ad. We do not know what role CD193^+^ NK cells have in the pathogenesis of the influenza virus. We may assume that these NK cells are involved in pathogenesis or, on the contrary, a reduced presence of these cells could have a positive effect on the course of the infection.

Since the surface marker F4/80 is found on several innate immune cells, including eosinophils, macrophages, and dendritic cells, it is very difficult to distinguish the specific phenotype of a subpopulation when detecting only one surface marker. However, according to several experimental studies, the surface markers CD45^+^ and F4/80^+^ are primarily expressed by eosinophils in Balb/c mice [60]. Eosinophils possess antiviral activity and may play a role in the innate immune response to influenza virus [61]. Interaction between IAV-exposed epithelial cells and eosinophils and cross-regulation promoted barrier responses to improve antiviral defenses in airway epithelial cells [62]. The frequency of F4/80^+^ CD45^+^ myeloid cells decreased after infection with WSN, NS80, and NS80ad. We suggest that a decreased level of F4/80^+^ CD45^+^ myeloid cells may contribute to the high pathogenicity of NS1-truncated viruses.

During the differentiation of active CD4^+^ Th lymphocytes, other subpopulations of cells arise that are essential in the regulation and effectiveness of adaptive immunity during influenza infection. The abundance of IL-12Rb2^+^ CD4^+^ T cells was increased only after infection with a lethal dose of WSN virus. A decreased number of CD193^+^ CD4^+^ T cells corresponds with previous findings and a decreased number of M2 macrophages [63,64].

We have also evaluated NKT cells with CD4 and NK1.1 receptors in the lungs of infected mice. The NKT cells participate in the cytotoxic responses of innate immunity [65]. In the lungs, NKT cells expressing CD4 and NK1.1 markers increased after infection with a lethal dose of influenza virus. It has been found that some NK1.1^+^ CD4^+^ T cells produce pro-inflammatory cytokines, which can either suppress or enhance the outcome of infection [66]. The expression of NK1.1^+^ cells does not affect the differentiation of CD4^+^ T lymphocytes into a specific phenotype of helper Th cells, as NK1.1^+^ CD4^+^ T cells exhibit properties of both Th1 and Tfh cell populations. Based on this knowledge, we can speculate that T lymphocytes go through a “transitional state” before differentiating into Th1 or Tfh cell populations [67]. The expression of NK1.1^+^ cells may define one of these “transitional states” [68]. In the lungs of infected mice, we also detected a subpopulation of NK1.1^+^ CD4^+^ T cells that expressed the IL-12Rb2 marker on their surface. The proportion of NKT cells with IL-12Rb2 increased in the lungs of mice after infection with a lethal dose of the virus. This subpopulation of cells has not yet been described, but we believe that it is induced by an increased concentration of IL-12 during lethal infection with IAVs. We can only speculate about the role of this subpopulation of NKT cells. Further investigation is needed to determine the function of these NKT cells.

Some limitations of this study merit consideration. A larger number of mice is needed to more precisely evaluate the accuracy of selected cell markers. The use of an LD100 dose of IAV did not allow us to monitor changes in cells in the innate and adaptive immune systems at specific time intervals. In the future, we would like to reduce the infectious dose to LD50 and attempt to monitor the immune response, for example, on days 2, 3, 4, and 5 after infection. We would also like to include receptors for distinguishing basophils, dendritic cells, and CD8 T cells.

## 4. Materials and Methods

### 4.1. Cells and Viruses

Vero cells (ATCC CCL81) were grown in Dulbecco’s modified Eagle’s medium (Lonza) containing 10% bovine serum (HyClone Laboratories, manufactured for Global Life Science, Pasching, Austria. The viruses A/WSN/33 (WSN) and a virus lacking the effector and C-terminal domains of the NS1 protein (NS80) were prepared using the reverse genetics system as previously described [22]. Both viruses were adapted to mice by five passages through the lungs as previously described [19]. All viruses were propagated in Vero cells.

To determine the 100% mouse lethal dose (LD100) of non-adapted (WSN and NS80) and adapted (WSNad and NS80ad) viruses, 10-fold serial dilutions of each virus were prepared, and groups of mice (*n* = 2) were infected intranasally with each dilution under Zoletil (5 mg/kg) anesthesia. The mice were monitored twice per day for weight loss and survival. The criteria for euthanasia were established by using the total score for observation of possible animal distress, such as weight loss exceeding 25% of the original body weight, decrease in appetite, weakness, shivering, depression, and moribund state of animals.

### 4.2. Mouse Experiments

Female BALB/c mice (4–5 weeks old; body weight approximately 19 g) were purchased from the Institute of Experimental Pharmacology and Toxicology, Toxicology and Animal Breeding, Dobrá Voda (SK). Groups of mice were anesthetized with Zoletil (5 mg/kg) and inoculated intranasally with non-lethal (10^2^ PFU of WSN, 10 PFU of WSNad, 10^4^ PFU of NS80, 10^2^ PFU of NS80ad) and lethal (10^4^ PFU of WSN, 10^2^ PFU of WSNad, 10^6^ PFU of NS80, 10^4^ PFU of NS80ad) doses of viruses in 40 µL PBS. Mock-inoculated with PBS served as controls. Mice were sacrificed via cervical dislocation on the third day post-infection, and the lungs were perfused with 50–100 mL of cold PBS. The lungs were aseptically harvested and processed for histological analyses, isolation of immune cells, or homogenized in 0.5 mL of PBS, and the aliquots of the organ homogenate were stored at −80 °C.

The mice were monitored daily for weight loss and survival. The criteria for euthanasia were established by using the total score for observation of possible animal distress. Weight loss exceeding 25% of the original body weight, decrease in appetite, weakness, shivering, depression, and moribund state of animals were monitored twice per day.

### 4.3. Determination of the Virus Titer

The supernatants from the infected lungs were used for virus quantification. The viral titers were determined as TCID50 titers using the endpoint dilution assay in Vero cells. The titers were calculated using the Reed–Muench method.

### 4.4. Histological Analysis

The collected lungs were processed according to standard histology procedures, fixed in 10% neutral-buffered formalin, and embedded in paraffin. Five-micrometer sections were stained with hematoxylin and eosin and examined via light microscopy.

### 4.5. RNA Sequencing and Gene Expression Analysis

Total RNA was extracted from the lung tissues of mice using the SV Total RNA Isolation System (Promega, Madison, WI, USA) according to the manufacturer’s instructions. RNA integrity was assessed using the QIAxpert system (Qiagen, Hilden, Germany). RNA sequencing via Illumina platforms, based on the mechanism of SBS (sequencing by synthesis), and standard bioinformatic analysis were performed by Novogene Co, Ltd., Cambridge, UK as described [19]) ClusterProfiler software and WebGestalt were used for enrichment analysis, including GO enrichment, DO enrichment, KEGG, Panther, and Reactome database enrichment. Hierarchical clustering of FPKm values was performed using Cluster 3.0, and heat maps were visualized in Java TreeView 3.0.

### 4.6. Immunophenotyping of Lung-Derived Immune Cells Using Multiparametric Flow Cytometry

The immune cells were isolated from lungs as described above. One hundred microliters of the cell suspension (1 × 10^6^ cells) were incubated with 10 μL of Fc receptor-blocking buffer (Innovex Biosciences Inc., Richmond, CA, USA) for 15 min. The cells were then immunolabeled with human/mouse IL-12 Rβ2 Alexa Fluor 488 (FAB1959G, R&D System, Minneapolis, MN, USA), PerCP/Cyanine 5.5 anti-mouse CD193 (CCR3) (BZ-144516, Biolegend, San Diego, CA, USA), Briliant Violet 421 anti-mouse IL-23R (BZ-150907, Biolegend), APC/cyanine7 anti-mouse CD68 (BZ-137024, Biolegend), PE/Cyanine7 anti-mouse Ly-6G (BZ-127618, Biolegend), PE anti-mouse CD163 (BZ-155308, Biolegend), APC anti-mouse F4/80 (BZ-123116, Biolegend), Briliant Violet 605 anti-mouse NK-1.1 (BZ-108753, Biolegend), BV650 BD OptiBuild TM rat anti-mouse CD4 (740479, Biosciences), and BUV496 BD OptiBuilt TM rat anti-mouse CD45 (749889, Biosciences) for 30 min at room temperature in the dark. Antibody concentration was 0.25–0.5 μg/sample. After labeling, cells were washed three times with flow cytometry staining buffer (1xPBS, 2% BSA, 2 mM EDTA, and 2 mM NaN_3_). The cells were analyzed using a FACS Aria Special Sorter (Becton Dickinson, Mountain View, San Jose, CA, USA) equipped with multiple laser systems. The obtained data were analyzed using De Novo FCS Express software (version 7.28.0035; De Novo Software, Los Angeles, CA, USA).

### 4.7. Flow Cytometric Gating Strategy and Controls

Mononuclear cells were first identified on FSC/SSC profiles, and doublets were excluded using FSC-H/FSC-W and SSC-H/SSC-W parameters. CD45^+^ leukocytes were then gated, and major immune subsets were defined using backbone markers: NK cells (CD45^+^NK1.1^+^CD4^−^), NK1.1^+^ on T cells (CD45^+^NK1.1^+^CD4^+^), CD4^+^ Th cells (CD45^+^CD4^+^NK1.1^−^), Ly-6G^+^ neutrophils (CD45^+^Ly-6G^+^), CD68^+^ macrophages (CD45^+^CD68^+^), and F4/80^+^ (CD45^+^F4/80^+^) myeloid cells. Within these backbone-gated populations, expression of additional markers was assessed to define functional/phenotypic subsets, including IL-12Rβ2, IL-23R, and CCR3 (CD193) on NK; NK1.1^+^ on T cells and CD4^+^ T cells; CD163, CCR3 (CD193), and F4/80 on Ly-6G^+^ neutrophils; and CD163, CCR3 (CD193), and F4/80 on CD68^+^ macrophages, as illustrated in Supplementary Figure S1. Compensation was calculated using single-stained mouse blood cells for each fluorochrome-conjugated antibody included in the panel (Supplementary Figure S2). In addition, fluorescence-minus-one (FMO) controls were used to establish negative and positive gating thresholds, enabling discrimination of true antigen-specific signal from background fluorescence. FMO controls were applied particularly to markers with low expression or poorly resolved positive populations, including IL-12Rβ2, IL-23R, and CCR3/CD193 (Supplementary Figure S3). All cell suspensions were prepared from freshly isolated mouse lungs and acquired on the same day; non-viable events were minimized via rapid processing and excluded based on abnormal FSC/SSC characteristics.

### 4.8. Statistical Analysis

Statistical analysis was performed by comparing the control group (noninfected mice, Mock) to infected mice or WSN-infected mice to NS80-infected mice. Data were analyzed using the unpaired Student’s t-test for data with two groups and ANOVA followed by Tukey’s post hoc test. *p* < 0.05 was statistically significant.

## 5. Conclusions

In this report, we characterized distinct subpopulations of innate immune cells in the lungs of mice infected with IAVs (Table 1). Although cytokine profiles differed in the lungs infected with wild-type viruses and viruses with truncated NS1 protein, all viruses affected the activity of the same immune cell subpopulations. The frequency of immune cells expressing Ly-6G, CD68, IL-23R, and IL-12Rb2 receptors was increased, whereas immune cells expressing CD163, F4/80, and CD193 on the surface decreased in the lungs of IAV-infected mice. The infectious dose of the virus influenced the distribution of cells detected among the subpopulations, while the NS1 protein did not affect the activity of subpopulations of detected immune cells. Each subpopulation appears to play a distinct role in innate immune responses after influenza infection, but we are still far from understanding how the subpopulations are activated and what roles they play in protecting against viruses and how they contribute to pathogenesis.

## Figures and Tables

**Table 1:** Summary of immune cell subpopulations in the lungs infected with influenza viruses.

	Ly-6G^+^	CD163^+^	F4/80^+^	CD193^+^	CD68^+^	IL-23R^+^	IL12Rβ2^+^	NK1.1^+^
**Neutrophils**	LD100↑	LD100↓	LD100↓					
**Macrophages**		↓	↓	↓	LD100↑			
**CD45^+^F4/80^+^ cells**			↓					
**NK cells**				↓		↑	LD100↑	
**NKT cells**							LD100↑	
**T cells**				↓			LD100↑	LD100↑

## Data Availability

The original contributions presented in this study are included in the article/Supplementary Material. Further inquiries can be directed to the corresponding author.

## Supplementary Materials

The following supporting information can be downloaded at: https://www.mdpi.com/article/10.3390/ijms27177522/s1.
