# Supplementary material for: Dose-Dependent Alterations in Lung Immune Subpopulations in Influenza a Virus Infection
Source: Int J Mol Sci. 2026 Aug 22;27(17):7522. doi: 10.3390/ijms27177522 (PMC13566561; doi:10.3390/ijms27177522)
Supplement: Supplementary file 1 [file ijms-27-07522-s001.zip › Legend for Supplementary Figures.pdf]

Supplementary Figure S1. Gating strategy for flow cytometric analysis of mouse lung immune cells. Mononuclear cells (MNCs) were first identified by FSC-A/SSC-A characteristics, and doublets were excluded using FSC-H vs FSC-W and SSC-H vs SSC-W gates. $CD45^+$ leukocytes were then gated, followed by definition of major immune subsets using backbone markers: NK cells ($CD45^+NK1.1^+CD4^-$), $NK1.1^+$ on T cells, $CD4^+$ Th cells ($CD45^+CD4^+NK1.1^-$), Ly-$6G^+$ neutrophils ($CD45^+$Ly-$6G^+$), $CD68^+$ macrophages ($CD45^+CD68^+$), and $F4/80^+$ ($CD45^+F4/80^+$) myeloid cells. Subsequent panels show the assessment of IL-12Rβ2, IL-23R, and CCR3 (CD193) on NK, $NK1.1^+$ on T cells, and $CD4^+$ T cells; CD163, CCR3 (CD193), and F4/80 expression on Ly-$6G^+$ neutrophils; and CD163, CCR3 (CD193), and F4/80 expression on $CD68^+$ macrophages. Color coding indicates the different backbone-gated populations and their corresponding phenotypic subsets, as summarized in the legend. Fluorescence minus one (FMO) controls were used to define the negative and positive gating thresholds for each marker. All samples were derived from freshly isolated mouse lungs and acquired on the same day without a dedicated viability dye; events with abnormal FSC/SSC profiles consistent with non-viable cells were excluded during gating.

Supplementary Figure S2. Compensation controls for the mouse 10-color flow-cytometry panel. Compensation was calculated using single-stained mouse blood cells for each fluorochrome-conjugated antibody included in the panel.

Supplementary Figure S3. Representative FMO controls for flow-cytometric gating of cytokine receptor-positive populations. FMO controls were used to define the negative population and establish positivity thresholds for IL-12Rβ2 (FITC), IL-23R (BV421), and CCR3/CD193 (PerCP-Cy5.5), which showed low expression or poorly resolved positive populations. Gates applied to the corresponding experimental samples were established based on these FMO controls.
