# Supplementary material for: Dose-Dependent Alterations in Lung Immune Subpopulations in Influenza a Virus Infection
Source: Int J Mol Sci. 2026 Aug 22;27(17):7522. doi: 10.3390/ijms27177522 (PMC13566561; doi:10.3390/ijms27177522)
Supplement: Supplementary file 1 [file ijms-27-07522-s001.zip › Supplementary Figure S1.pdf]

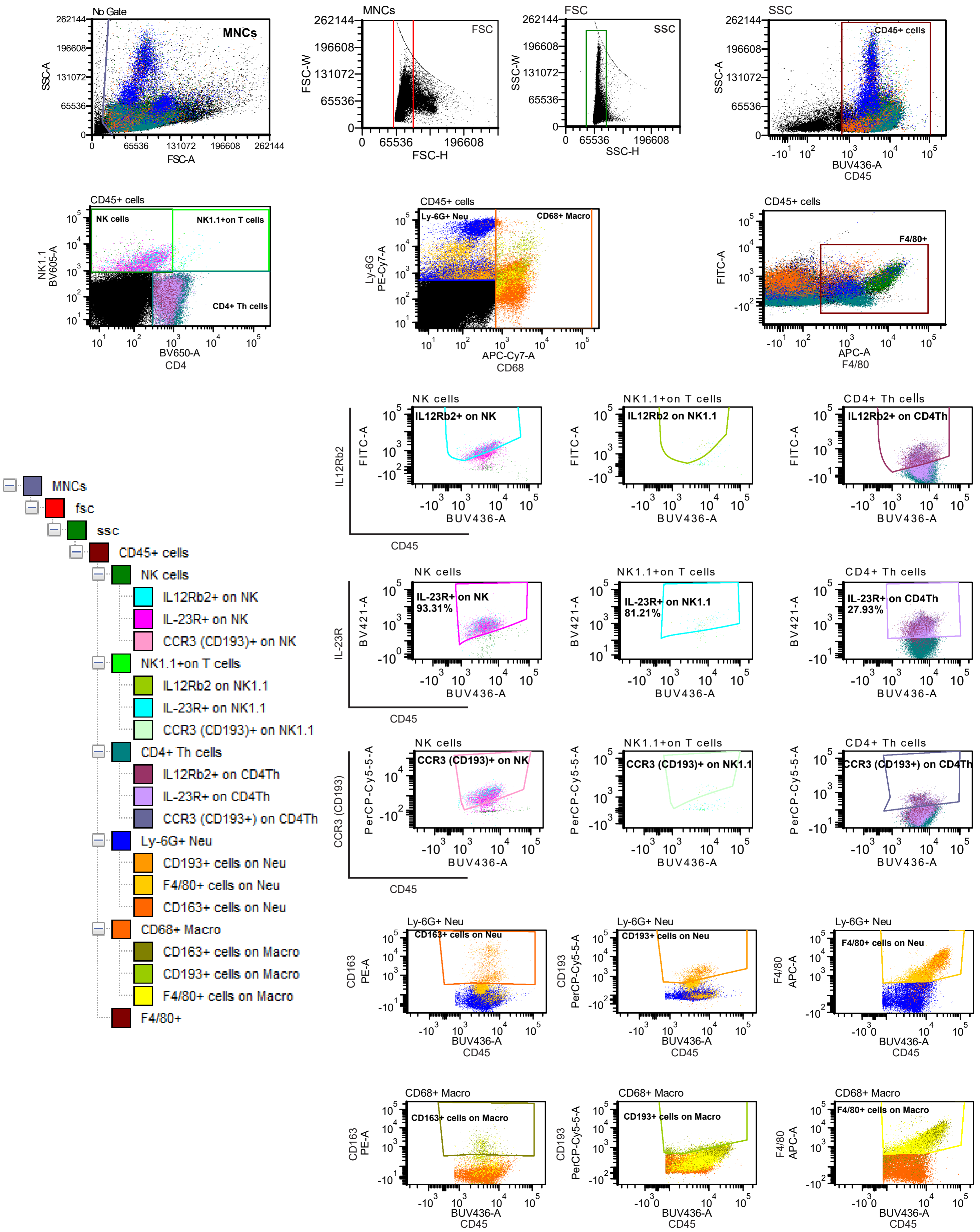

No Gate
MNCs
SSC-A
FSC-A
FSC
FSC-W
FSC-H
SSC
SSC-W
SSC-H
CD45+ cells
BUV436-A
CD45
NK cells
NK1.1+on T cells
CD4+ Th cells
NK1.1
BV605-A
BV650-A
CD4
Ly-6G+ Neu
CD68+ Macro
Ly-6G
PE-Cy7-A
APC-Cy7-A
CD68
F4/80+
FITC-A
APC-A
F4/80
IL12Rb2
IL12Rb2+ on NK
IL12Rb2 on NK1.1
IL12Rb2+ on CD4Th
IL-23R
BV421-A
IL-23R+ on NK
93.31%
IL-23R+ on NK1.1
81.21%
IL-23R+ on CD4Th
27.93%
CCR3 (CD193)
PerCP-Cy5-5-A
CCR3 (CD193)+ on NK
CCR3 (CD193)+ on NK1.1
CCR3 (CD193+) on CD4Th
CD163
PE-A
CD163+ cells on Neu
CD193
CD193+ cells on Neu
F4/80+ cells on Neu
CD163+ cells on Macro
CD193+ cells on Macro
F4/80+ cells on Macro
MNCs
fsc
ssc
CD45+ cells
NK cells
IL12Rb2+ on NK
IL-23R+ on NK
CCR3 (CD193)+ on NK
NK1.1+on T cells
IL12Rb2 on NK1.1
IL-23R+ on NK1.1
CCR3 (CD193)+ on NK1.1
CD4+ Th cells
IL12Rb2+ on CD4Th
IL-23R+ on CD4Th
CCR3 (CD193+) on CD4Th
Ly-6G+ Neu
CD193+ cells on Neu
F4/80+ cells on Neu
CD163+ cells on Neu
CD68+ Macro
CD163+ cells on Macro
CD193+ cells on Macro
F4/80+ cells on Macro
F4/80+
