# Supplementary material for: Dose-Dependent Alterations in Lung Immune Subpopulations in Influenza a Virus Infection
Source: Int J Mol Sci. 2026 Aug 22;27(17):7522. doi: 10.3390/ijms27177522 (PMC13566561; doi:10.3390/ijms27177522)
Supplement: Supplementary file 1 [file ijms-27-07522-s001.zip › Supplementary Figure S2.pdf]

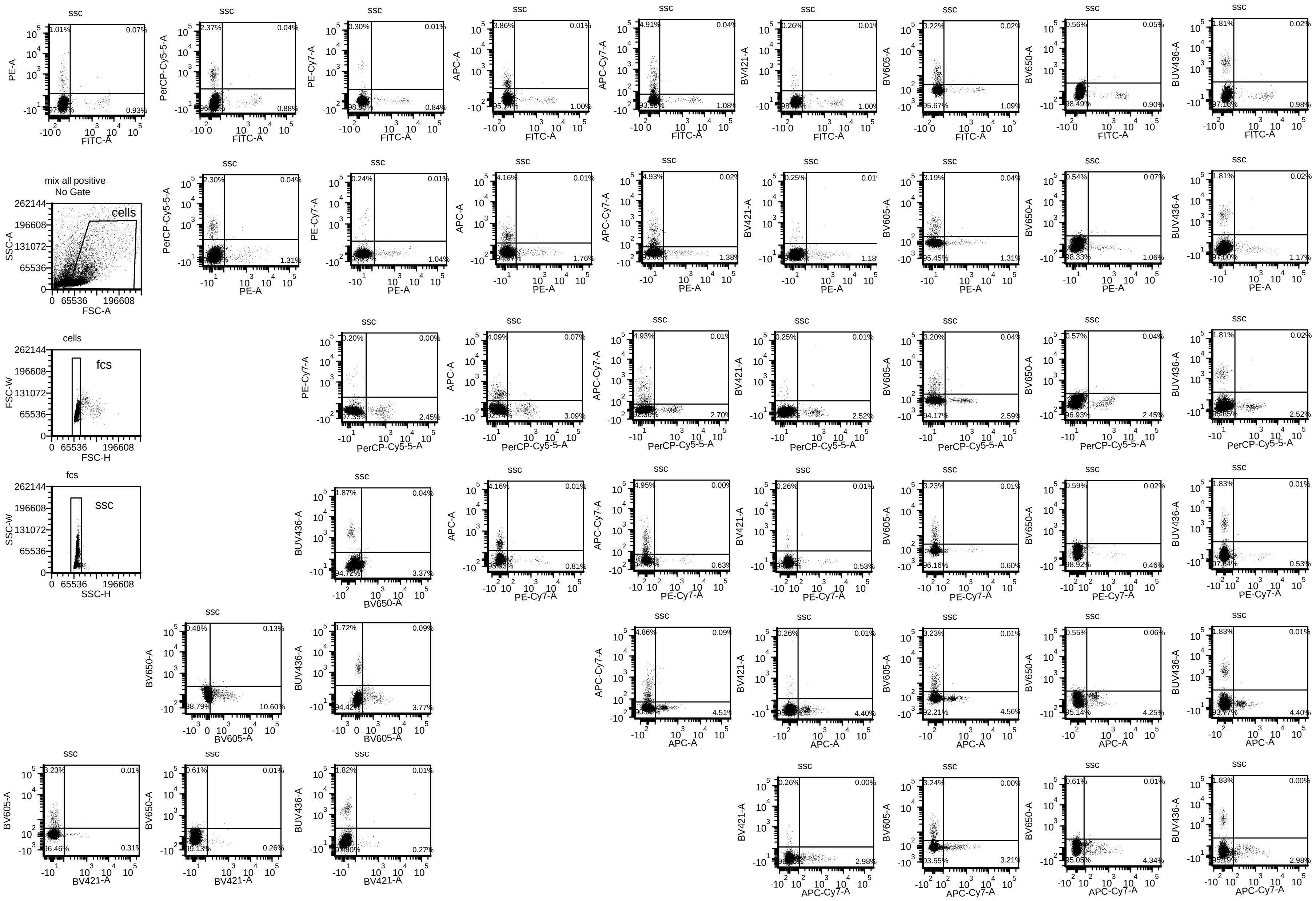

mix all positive
No Gate
cells
fcs
ssc
SSC-A
FSC-A
FSC-W
FSC-H
SSC-W
SSC-H
FITC-A
PE-A
PerCP-Cy5-5-A
PE-Cy7-A
APC-A
APC-Cy7-A
BV421-A
BV605-A
BV650-A
BUV436-A
