# Supplementary material for: Dose-Dependent Alterations in Lung Immune Subpopulations in Influenza a Virus Infection
Source: Int J Mol Sci. 2026 Aug 22;27(17):7522. doi: 10.3390/ijms27177522 (PMC13566561; doi:10.3390/ijms27177522)

FMO FITC

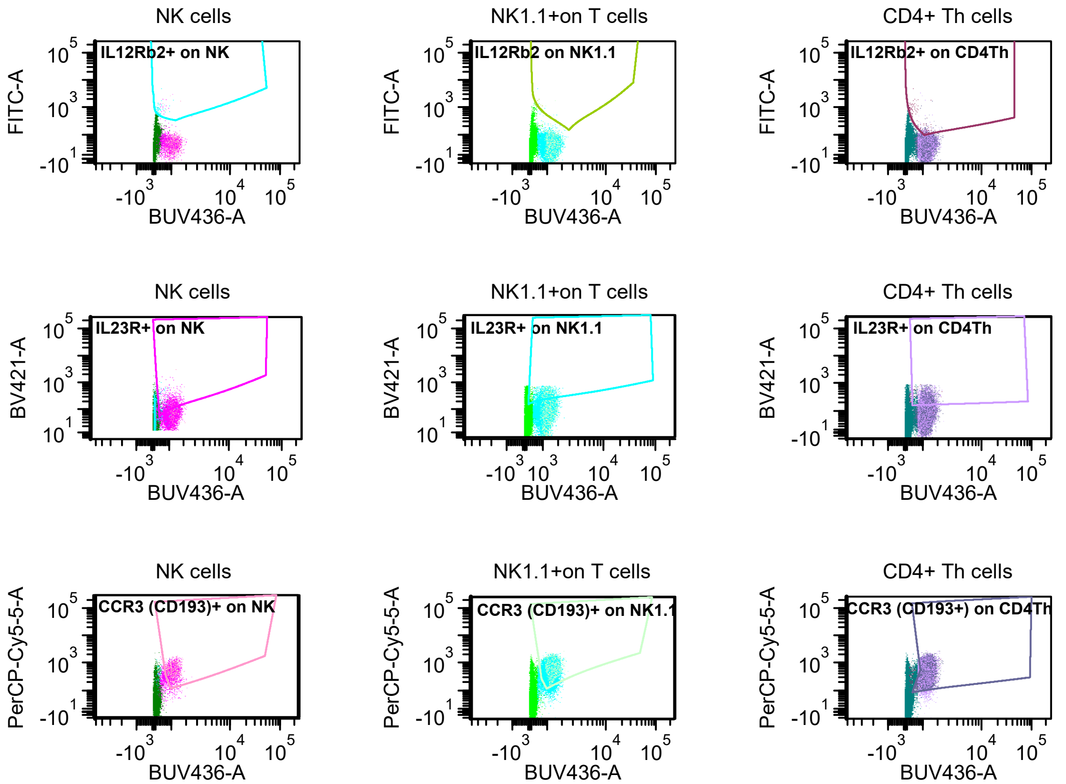
NK cells
NK1.1+on T cells
CD4+ Th cells
IL12Rb2+ on NK
IL12Rb2 on NK1.1
IL12Rb2+ on CD4Th
FITC-A
IL23R+ on NK
IL23R+ on NK1.1
IL23R+ on CD4Th
BV421-A
CCR3 (CD193)+ on NK
CCR3 (CD193)+ on NK1.1
CCR3 (CD193+) on CD4Th
PerCP-Cy5-5-A
BUV436-A

FMO BV421

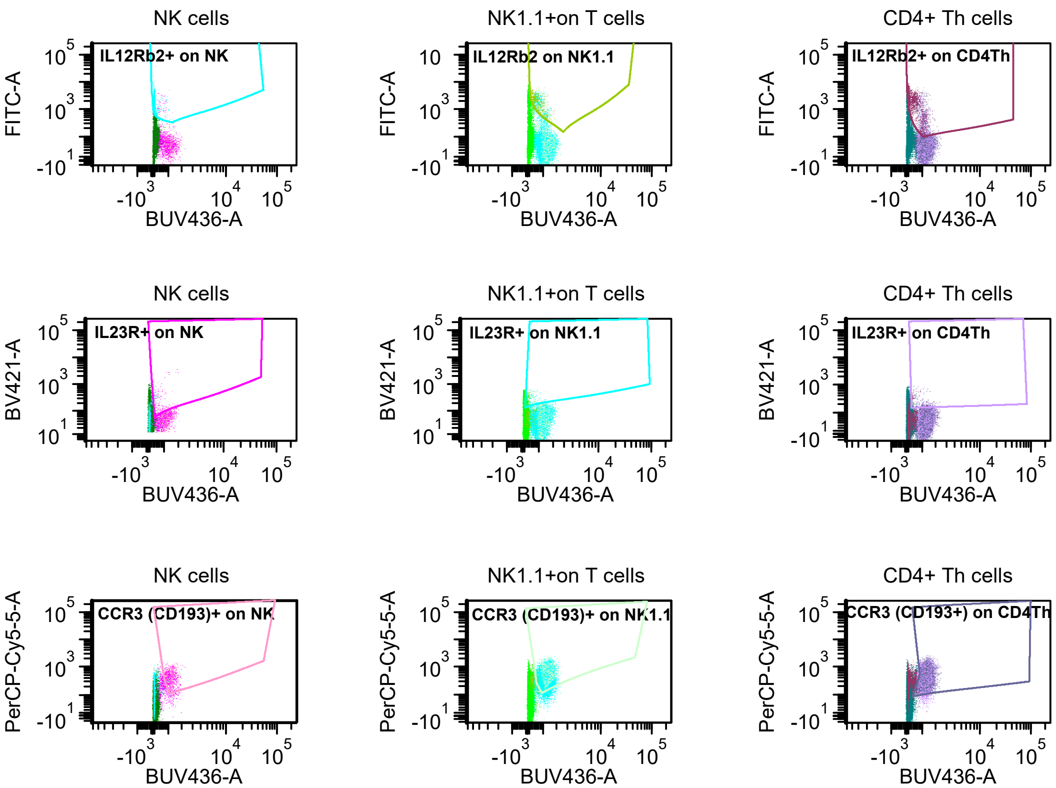

NK cells
NK1.1+on T cells
CD4+ Th cells
IL12Rb2+ on NK
IL12Rb2 on NK1.1
IL12Rb2+ on CD4Th
FITC-A
IL23R+ on NK
IL23R+ on NK1.1
IL23R+ on CD4Th
BV421-A
CCR3 (CD193)+ on NK
CCR3 (CD193)+ on NK1.1
CCR3 (CD193+) on CD4Th
PerCP-Cy5-5-A
BUV436-A

FMO PerCP-Cy5-5.5

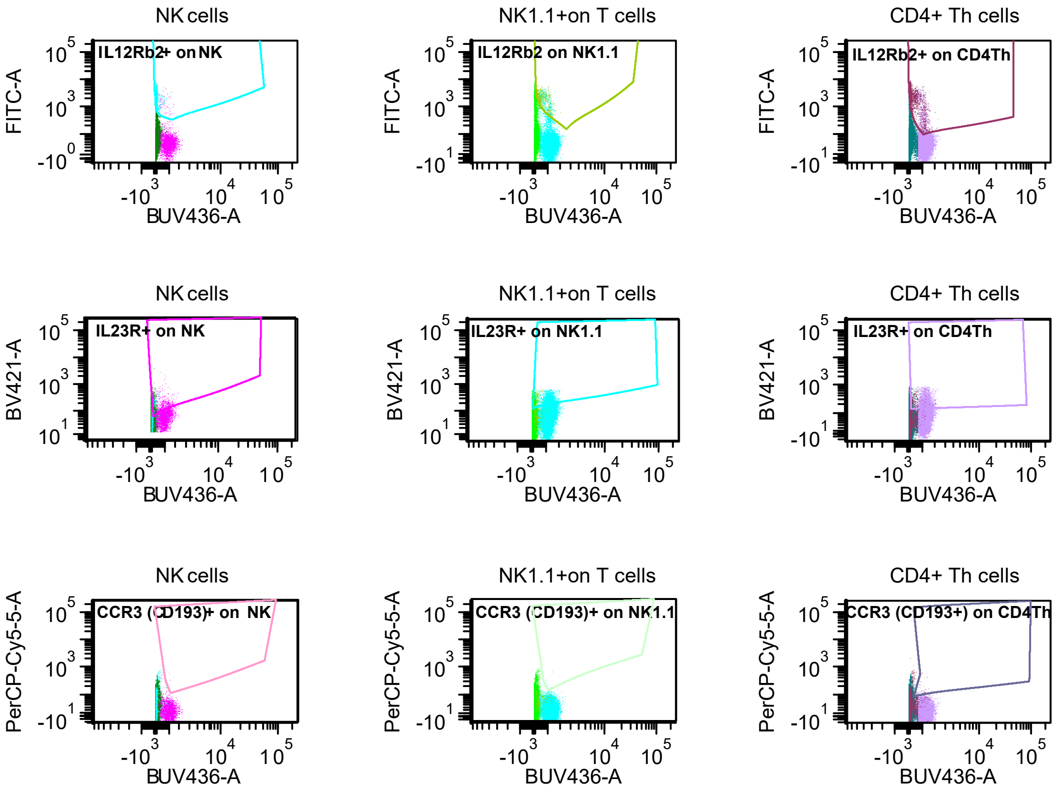

Supplement: Supplementary file 1 [file ijms-27-07522-s001.zip › Supplementary Figure S3.pdf]
